# Laparoscopic extraction of a symptomatic upper abdominal pedunculated parietal peritoneal lipoma arising intermittent abdominal pain: a case report

**DOI:** 10.1186/s13256-024-04511-5

**Published:** 2024-04-22

**Authors:** Yu Dohmoto, Yoshimasa Akashi, Koichi Ogawa, Tsuyoshi Enomoto, Yusuke Ohara, Yohei Owada, Tatsuya Oda

**Affiliations:** Department of Gastrointestinal and Hepato-Biliary-Pancreatic Surgery, Faculty of Medicine, University of Tsukuba, 1-1-1 Tennodai, Tsukuba, Ibaraki 305-8575 Japan

**Keywords:** Abdominal wall, Lipoma, Peritoneum

## Abstract

**Introduction:**

Lipomas arising in the parietal peritoneum are rare, and some of them cause abdominal pain due to torsion of the pedunculated peritoneum. We encountered a case of parietal peritoneal lipoma arising upper peritoneum. In this report, we describe the detail of clinical presentation and discuss its potential pathogenesis and treatment strategy.

**Case presentation:**

45 year-old Japanese female patient presented with long-lasting intermittent pain in the left upper abdominal region. Abdominal imaging showed a well-defined fatty mass measuring 40 mm in size, suggesting a parietal peritoneal lipoma. Laparoscopy revealed a tumor with a twisted peduncle; however, no adhesion of the surrounding tissues and ischemic changes were visible. The tumor was easily removed by dissection of the tumor pedicle.

**Conclusion:**

Parietal peritoneal lipoma often shows pedunculated form and it causes abdominal pain by the torsion of tumor pedicle. Therefore, this type of lipoma should be considered a more aggressive surgery.

## Introduction

Lipomas are common benign tumors that can occur in any part of the body; however, those arising in the parietal peritoneum are rare [1]. Some of these lipomas are pedunculated and cause abdominal pain due to torsion of the attaching peritoneum [2]. The most frequent locations of these pain-causing lipomas are the right iliac fossa, mimicking the clinical appearance of appendicitis [1–3], and any other lipomas are located in the mid or lower abdomen [4–9].

We herein present the first case of pedunculated lipoma arising in the upper parietal peritoneum.

## Case presentation

A 45-year-old Japanese woman presented with intermittent abdominal pain in the left upper quadrant for more than 5 months. Two months after the onset of symptoms, the patient visited a clinic and was treated with medication; however, symptoms recurred two months later. A 40 mm well-demarcated low-density mass was detected on computed tomography (CT) scan, and she was referred to our hospital for further examination.

**Fig. 1:**
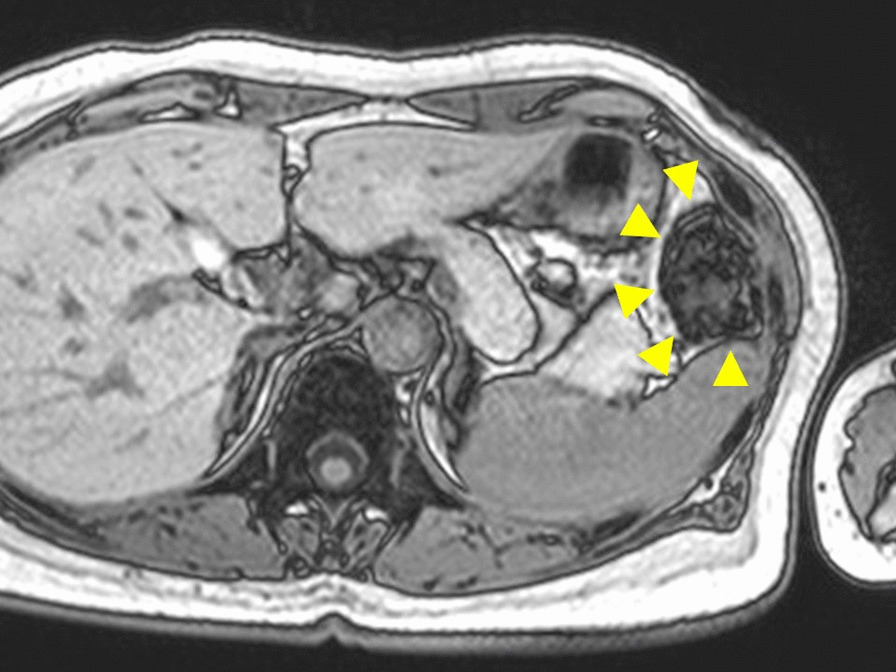
Magnetic resonance imaging of the tumor. Magnetic resonance imaging examination with contrast material shows a pedunculated tumor that attaches to the parietal peritoneum (arrowheads)

She had no other symptoms, such as fever, nausea or vomiting, urinary or defecation disorders, and had no significant past medical history and abdominal laparotomy. Laboratory examination revealed no specific abnormal findings. Contrast-enhanced magnetic resonance imaging (MRI) revealed that the mass was a non-enhanced fatty tissue and seemed to have a pedicle attached to the parietal peritoneum (Fig. 1). Atypical lipomatous tumor or well differentiated liposarcoma were considered as differential diagnosis; however, benign lipoma was strongly suggested by the imaging findings.

Although a benign lipoma was strongly suspected, the tumor was considered to have caused her intermittent abdominal pain because no other abnormalities were present. Therefore, we decided to perform a laparoscopic tumor resection.

**Fig. 2:**
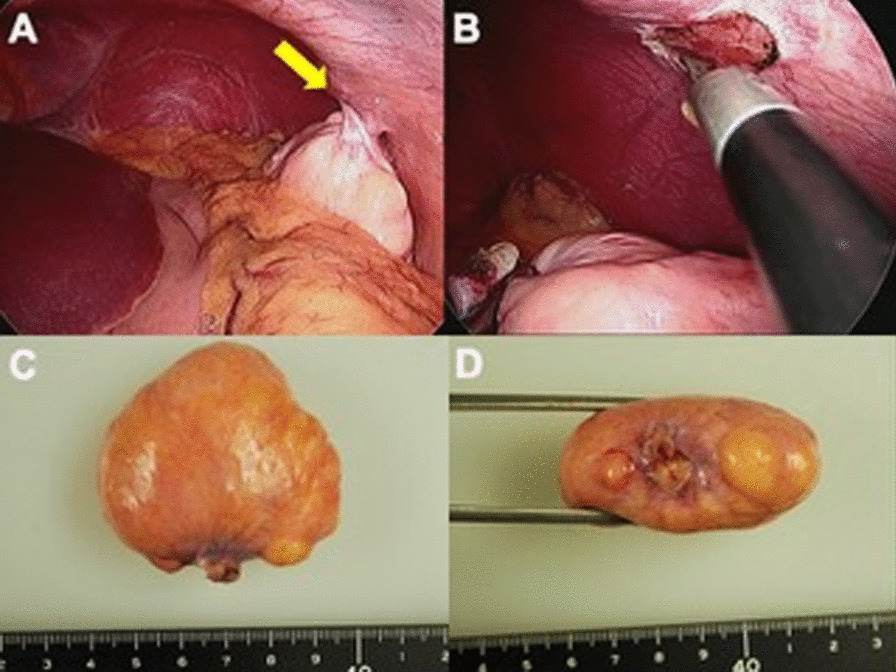
Intraoperative and macroscopic findings of the tumor. The pedunculated tumor of the left upper parietal peritoneum is observed in laparoscopy and its pedicle was twisted (**A**, arrow). The tumor was attached only to peritoneum and had no continuity to the muscle or vessels (**B**). Macroscopic findings of tumor (**C** tumor surface, **D** stump of pedicle). A soft, whitish-yellow, smooth surface mass with a narrow pedicle. No evidence of congestion, ischemia, or acute hemorrhage

The operation was performed with four-port trocars: a 12-mm trocar at the umbilicus, three 5-mm trocars at the right and left hypochondriac and right lumbar regions. The tumor was observed as a lipomatous, smooth, and whitish-yellow surface lesion attached to the parietal peritoneum in the left hypochondriac region with a narrow pedicle (Fig. 2A). The pedicle was twisted; however, no ischemic changes were observed in the tumor. There was no adhesion of the tumor to any other organs or the greater omentum. The tumor was easily removed only by dissecting the pedicle and extracted from the umbilical port after insertion of a retrieval bag (Fig. 2B).

The patient recovered without any complications and was discharged on postoperative day 2. The resected tumor was 50 × 45 × 20 mm in size, and histological findings showed diffuse proliferation of mature adipocytes with collagen fibers and was finally diagnosed as fibrolipoma (Fig. 2C, D). The abdominal pain was relieved after surgery without recurrence of abdominal symptoms during the one-year follow-up period.

## Discussion

Parietal peritoneal lipomas are rare, and only nine cases have been reported to date (Table 1) [1–9]. Among these cases, four presented with torsion of pedunculated lipoma, in which patients presented with right lower abdominal pain mimicking acute appendicitis [1–3, 7]. Those cases showed aggravated pain, and signs of peritonitis were present on physical examination. In all of these cases, congested, ischemic, or acute hemorrhagic lipomas with twisted torsion peduncle were observed, and then ischemic change itself or torsion of the peritoneum was considered as the cause of pain aggravation. In our case, the patient’s chief complaint was intermittent abdominal pain in the left upper quadrant region, and as opposed to previous cases, the pain did not worsen with time, and signs of peritonitis were not present. Intraoperatively, the peduncle of the lipoma was twisted; however, the lipoma did not show signs of ischemia, congestion, or hemorrhage. This may explain why the pain was not aggravated in contrast to other reported cases.

**Table 1:** Reports regarding the treatment of the parietal peritoneal lipoma

No.	References (year)	Age	Sex	Location	Maximum diameter (cm)	Torsion of pedicle	Symptom	Diagnosis	Treatment
1	Barut (2006) [5]	67	Female	Median umbilical ligament	6	−	Abdominal pain, nausea	Laparotomy	Laparotomy
2	Bunker (2013) [2]	34	Female	Right iliac fossa	n.a	+	Abdominal pain	Laparoscopy	Laparoscopy
3	Bang (2014) [4]	75	Male	Right iliac fossa	6.5	−	Abdominal pain	CT	Laparotomy
4	Shrestha (2014) [1]	32	Male	Right iliac fossa	3	+	Abdominal pain	Laparoscopy	Laparoscopy
5	Sathyakrishna (2014) [3]	21	Female	Right iliac fossa	n.a	+	Abdominal pain	Laparoscopy	Laparoscopy
6	Salgaonkar (2016) [9]	79	Male	Left iliac fossa	6.3	−	Abdominal pain	CT	Laparoscopy
7	Özemir (2016) [7]	35	Female	Right iliac fossa	4	+	Abdominal pain	Laparoscopy	Laparoscopy
8	Choi (2018) [6]	36	Male	Right iliac fossa	22	–	Urinary frequency	CT	Laparoscopy
9	Pillay (2021) [8]	62	Male	Median umbilical ligament	2	−	Abdominal pain	US	Laparoscopy
10	Present case (2022)	45	Female	Left upper peritoneum	5	+	Abdominal pain	CT, MRI	Laparoscopy

*n.a.* not available

*US* ultrasound, *CT* computed tomography, *MRI* magnetic resonance imaging

In the previous nine cases of parietal peritoneal lipomas, six (66.7%) were located in the right iliac fossa and appeared around the right lateral umbilical fold. Additionally, two (22.2%) of those developed from the median umbilical fold or falciform ligament, and the remaining one developed around the left iliac fossa. In the umbilical folds, fat tissues generally exist around the umbilical cord or inferior epigastric vessels, and the amounts are relatively higher than in other areas of the peritoneum, excluding the retroperitoneum, which includes the thick fat pad of Gerota. Lipomas arising from the retroperitoneum are not rare in the parietal peritoneum; therefore, the incidence of lipomas and the amount of fat tissues in each location appears to be proportional. If so, all nine previous parietal lipomas located in the mid or lower abdomen, particularly around the umbilical folds, are understandable. Furthermore, some cases reported as preperitoneal lipomas around inguinal areas may be included in the same pathogenic entities [10]. Although this case is included in the disease concept of epiploic appendagitis, this disease is usually recognized as occurring in the epiploic appendices. It should be kept in mind that it can also occur in appendices originating in epigastric abdominal wall as in our case[11]. From this point of view, our case is extremely rare and there is no previous case report of parietal peritoneal lipoma arising in the upper side of the parietal peritoneum as far as we researched. The location is not generally observed in rich preperitoneal fat tissues; therefore, ectopic or misplaced adipose tissue is considered a possible cause for this case.

As lipomas are benign tumors with low potential for malignant transformation, in the abdominal cavity they may be observed without excision and can be detected accidentally during intraoperative procedures, particularly in cases that are not accompanied by any symptoms. In some reports, lipomas accidentally found during intraabdominal operation were left unexcised because there was no evidence of contributing to the patient’s symptoms [8, 10]. The pedunculated form of parietal lipoma should be considered a more aggressive surgery owing to the risk of its torsion. The operation of pedunculated lipoma exclusion is quite simple and easy, as in our case; just the resected pedicle of lipoma, and it appears to be a better indication for a minimally invasive approach. It was difficult to diagnose preoperatively in five out of nine cases, and diagnosed by explorative laparoscopy or laparotomy. Therefore, in cases of abdominal symptoms with or without a definitive diagnosis, laparoscopic surgery appears to be a better treatment strategy.

## Conclusion

Differential diagnoses of lipoma by imaging exams is not so complex, and asymptomatic lipoma can be observed without intervention. However, pedunculated form is often observed in lipomas arising in parietal peritoneum, and it cause abdominal pain by the torsion of tumor pedicle. Therefore, this type of lipoma should be considered a more aggressive surgery.

## Data Availability

The data that support the findings of this study are available from the corresponding author upon reasonable request.

## References

[1] Shrestha BB, Karmacharya M. Torsion of a lipoma of parietal peritoneum: A rare case mimicking acute appendicitis. *J Surg Case Reports* 2014; **2014**: rju062-rju062. 10.1093/jscr/rju062 PMC4062179 24941942

[2] Bunker DLJ, Ilie VG, Halder TK (2013). Torsion of an abdominal-wall pedunculated lipoma: a rare differential diagnosis for right iliac fossa pain. Case Reports in Surgery.

[3] Sathyakrishna BR, Boggram SG, Jannu NR (2014). Twisting lipoma presenting as appendicitis—a rare presentation. J Clin Diagn Res.

[4] Bang CS, Kim YS, Baik GH, Han SH (2014). A case of lipoma of parietal peritoneum causing abdominal pain. Korean J Gastroenterol.

[5] Barut I, Tarhan OR, Cerci C, Ciris M, Tasliyar E (2006). Lipoma of the parietal peritoneum: an unusual cause of abdominal pain. Ann Saudi Med.

[6] Choi H, Ryu D, Choi JW, Xu Y, Kim Y (2018). A giant lipoma of the parietal peritoneum: laparoscopic excision with the parietal peritoneum preserving procedure - A case report with literature review. BMC Surg.

[7] Özemir İA, Orhun K, Bilgiç Ç *et al.* [Torsion of a preperitoneal pedunculated lipoma of anterior abdominal wall mimicking acute appendicitis]. Ulusal Travma ve Acil Cerrahi Dergisi 2016; **22**: 502-504. 10.5505/tjtes.2016.63500 27849330

[8] Pillay Y. Parietal peritoneal lipomas: a first case report of two lipomas of the parietal peritoneum. J Surg Case Reports 2021; 2021: rjab162. 10.1093/jscr/rjab162 PMC8096476 33976760

[9] Salgaonkar HP, Behera RR, Katara AN, Bhandarkar DS (2016). Laparoscopic excision of a lipoma of parietal peritoneum. J Minimal Access Surg.

[10] Nishida K, Ochiai S, Lefor AK (2022). A “sacless hernia” with the orifice obscured by a preperitoneal lipoma: a case report. Int J Surg Case Rep.

[11] Dario G, Roberto C, Giovanni C (2019). CT imaging findings of epiploic appendagitis: an unusual cause of abdominal pain. Insight Imaging.

